# 3D-printed indirect bonding trays and transfer jigs for lingual brackets: Digital workflows and two case reports

**DOI:** 10.1016/j.heliyon.2024.e32035

**Published:** 2024-05-28

**Authors:** Viet Anh Nguyen

**Affiliations:** Faculty of Dentistry, Phenikaa University, Yen Nghia, Ha Dong, Hanoi, 12116, Viet Nam

**Keywords:** Lingual appliances, Bracket transfer jigs, Indirect bonding tray, 3D printing, Case report

## Abstract

With the advancement of 3-dimensionally (3D) printing technology, orthodontists can design and fabricate 3D-printed indirect bonding trays and transfer jigs for lingual brackets independently from the laboratory. The present article describes, in detail, the digital workflows for designing and fabricating 3D-printed lingual bracket indirect bonding trays and transfer jigs. Additionally, it aims to demonstrate the effectiveness of this approach in managing common orthodontic issues in adult patients. The first case report exemplifies the successful management of moderate crowding in a Class I adult patient using a non-extraction approach with lingual brackets and flexible 3D-printed indirect bonding trays. The second case illustrates the application of lingual brackets and rigid 3D-printed indirect bonding trays in managing a skeletal Class II adult patient with mouth protrusion requiring four-bicuspid extractions. The achieved good treatment results might demonstrate the high transfer accuracy of 3D-printed lingual bracket indirect bonding trays. Additional studies with large sample sizes should be conducted to compare the effectiveness and efficiency of 3D-printed trays with other tray types.

## 1 Introduction

Accuracy of bracket position is a key to successful orthodontic outcomes. Unlike labial orthodontics, direct bonding is challenging in lingual orthodontics due to the variability and complexity of lingual tooth surface anatomy, along with difficulties in intraoral visualization and measurement of bracket positions. Therefore, indirect bonding offers significant advantages in lingual orthodontics, providing greater precision and control over the process [1,2]. In the analog laboratory technique, transfer jigs are fabricated after positioning lingual brackets on setup models with ideal tooth alignment [3,4]. This analog technique is complex, time-consuming, and requires experienced technicians to incorporate prescription and overcorrection values into the lingual bracket positions.

With the advancement of digital technology in orthodontics, indirect bonding trays for labial brackets can be designed and 3-dimensionally (3D) printed directly without the intermediate step of fabricating setup models [5,6]. However, 3D-printed transfer jigs or bonding trays for lingual brackets are yet to be designed with most commercial orthodontic software due to the limited popularity of lingual appliances compared to labial ones. Alternatively, vacuum-formed indirect bonding trays can be created using 3D-printed malocclusion models with lingual brackets or individual transfer jigs made using flowable composites and 3D-printed setup models with lingual bracket guides [7,8]. However, these approaches involve additional steps, making them more time-consuming and potentially prone to errors.

The present article describes, in detail, the digital workflows for designing and fabricating 3D-printed lingual bracket indirect bonding trays and transfer jigs. Furthermore, it aims to demonstrate the effectiveness of this approach in managing common orthodontic issues in adult patients. The first case report showcases the successful treatment of moderate crowding in a Class I adult patient using a non-extraction approach with lingual brackets and flexible 3D-printed indirect bonding trays. The second case illustrates the application of lingual brackets and rigid 3D-printed indirect bonding trays in treating a skeletal Class II adult patient with mouth protrusion requiring four-bicuspid extractions.

## 2 Design and fabrication

### 2.1 Virtual setup model construction

**Fig. 1:**
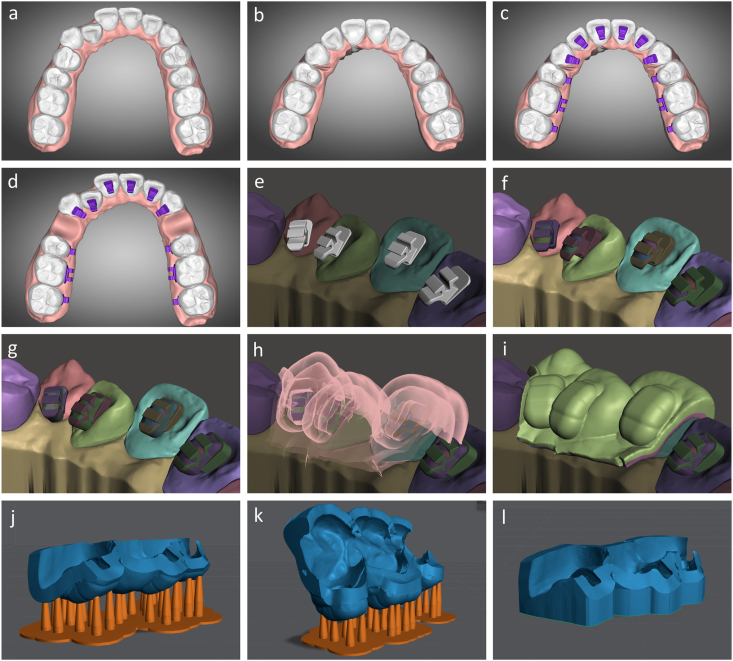
(a) Tooth and gingiva segmentation. (b) Virtual orthodontic setup. (c) Lingual bracket placement. (d,e) Lingual brackets are moved back to the initial malocclusion state. (f) Bracket bases are extruded. (g) Bracket undercuts are blocked out. (h) Creation of the inner and outer tray layer using ‘offset’ commands. (i) Formation of the tray using a ‘join’ command. (j) 3D printing partially enclosed trays. (k) 3D printing fully enclosed trays. (l) 3D printing flexible trays.

The digital impression of the patient's dentition is imported into orthodontic software (Autolign, Diorco, Korea). The teeth and gingiva are segmented by selecting each tooth's mesial and distal points (Fig. 1a). Next, an orthodontic setup is created with ideal tooth alignment according to the treatment plan with integrated prescription and overcorrection values of the first, second, and third orders (Fig. 1b). The arch form and occlusal plane are displayed to facilitate the leveling and symmetric alignment. The panoramic radiograph should be consulted to determine the root inclination.

### 2.2 Virtual lingual bracket positioning

The virtual lingual bracket positioning is based on the same concept as the analog lingual laboratory technique in which the brackets are positioned with the guidance of a full-size archwire on the setup model (Fig. 1c). The virtual archwire's shape and position are adjusted so that the bracket-tooth distances are as close as possible, but no smaller than 0.03 mm to ensure sufficient space for orthodontic adhesives. Lingual archwires are typically straight, but in cases of missing incisors or canines, some offset bends may be necessary to compensate for tooth size differences. Lingual brackets are typically centered on each tooth mesiodistally. However, in cases of crowding, they may need to be moved slightly mesially or distally to prevent impingement by other teeth or brackets, allowing them to be bonded to the initial malocclusion dentition.

### 2.3 Indirect bonding tray and transfer jig design

When the bracket positions are determined on the corresponding teeth on the ideal setup models, the teeth and attached lingual brackets are virtually moved back to the initial misalignment state (Fig. 1d). These initial models with accompanying lingual brackets are exported and imported into free 3D design software (Meshmixer, Autodesk, USA, and Medit Design, Medit, Korea) to design the indirect bonding trays and transfer jigs (Fig. 1e).

First, the bracket bases are extruded to fill the space between the brackets and lingual tooth surfaces (Fig. 1f). Then, all bracket slots and undercuts below the bracket wings are blocked out (Fig. 1g). Next, a Boolean union command is applied to combine all data into one mesh file. The inner and outer surface of the indirect bonding tray is created using an ‘offset’ command with a distance of 0.03 mm and 1.53 mm from the tooth and bracket surfaces, respectively (Fig. 1h). By using a ‘join’ command, an indirect bonding tray of 1.5 mm thickness is created (Fig. 1i).

The bracket lodgement design can fully or partially enclose the bracket depending on the orthodontist's preference. If the partially enclosed design is chosen, the gingival wall of the lodgement is trimmed on the software. In cases of severe crowding preventing the initial bonding of some teeth due to impingement, individual transfer jigs are designed with the same procedures but based on the ideal setup models instead of the initial malocclusion ones.

### 2.4 Indirect bonding tray and transfer jig 3D printing

The designs of the indirect bonding trays and transfer jigs are exported and imported into a slicer software (Lychee Slicer, Mango 3D, France) for support placement and slicing. The trays with partially enclosed lodgement designs are printed with a build angle of 180° to minimize printing time (Fig. 1j) [9]. However, when the fully enclosed design is chosen, the trays have to be segmented into shorter spans to be printed with a build angle of approximately 110° so that the gingival wall is adequately supported during 3D printing (Fig. 1k).

The indirect bonding trays can be printed with a rigid (Surgical Guide, Ludent, Korea) or flexible resin (Ortho IBT, Ludent, Korea). When a rigid resin is chosen, spans of no more than three teeth should be grouped to avoid tray dislodgement during bonding due to post-curing shrinkage. When a flexible resin is used, the trays should be printed directly on the printer's platform without support by designing a flat base with an ‘extrude down’ command (Fig. 1l). The thickness of flexible trays should be 2.0 mm. The slicing file is exported and added to a resin 3D printer with stereolithography, digital light processing, or liquid crystal display technology.

**Fig. 2:**
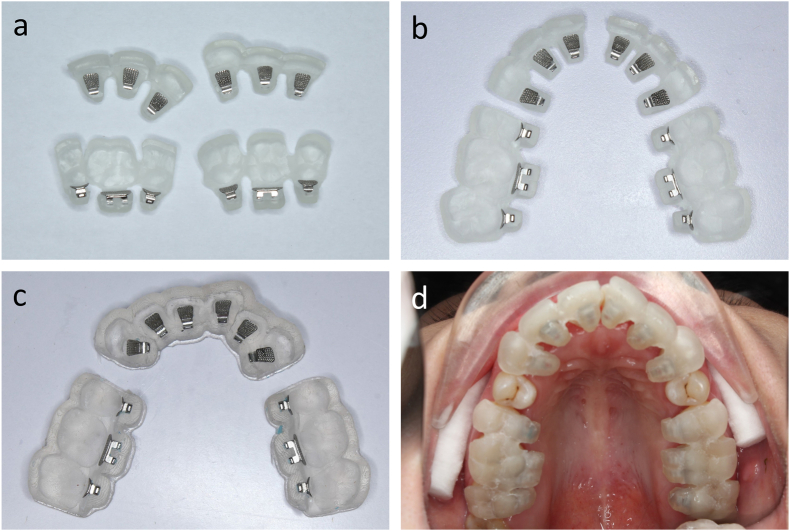
(a) Fully enclosed indirect bonding trays with inserted lingual brackets. (b) Partially enclosed trays. (c) Flexible trays. (d) Tray placement in vivo.

After printing, the indirect bonding trays are removed from the platform, cleaned with isopropyl alcohol, and post-cured with an ultraviolet lamp. With rigid trays, all printing supports are removed and the trays are trimmed with burs to allow easier removal during clinical bonding. Finally, lingual brackets are manually inserted into the trays (Fig. 2a–c). Study models are also 3D-printed for tray try-in.

### 2.5 Archwire forming

**Fig. 3:**
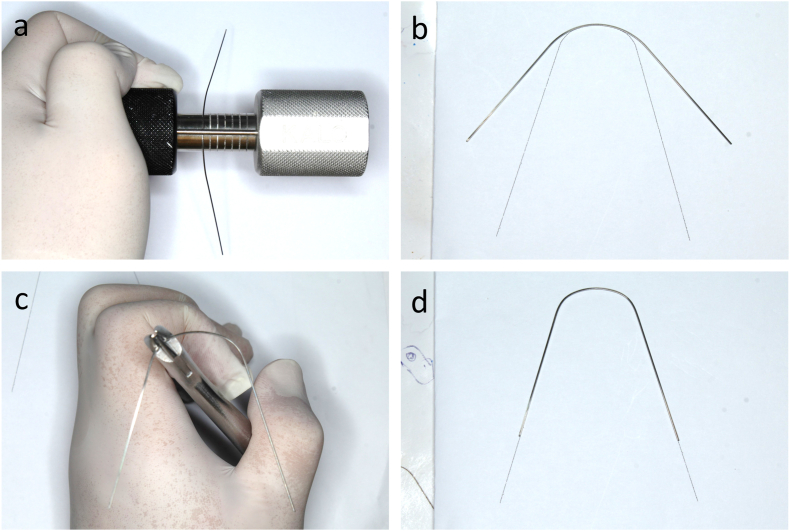
Customized lingual straight archwire forming. (a) Formation of the anterior curve. (b) Adaption to the patient's form. (c) Formation of the archwire angle. (d) Completed customized lingual straight archwire.

Lingual straight archwires were used to simplify the archwire forming procedures according to the concept of Scuzzo and Lombardo [[10], [11], [12]]. The patient's individual archwire template is exported from the software and printed on plain paper. Next, a lingual turret is used to form the anterior curve of the archwire from a stainless steel straight wire followed by adjusting the angle of the archwire using a three-prong plier or a hollow-chop plier until matching the archwire template (Fig. 3a–d). These stiff individual archwires are used in the space closure stage and finishing stage. However, stock nickel-titanium archwires are used in the leveling and alignment stage because these flexible archwires are hard to adjust and have limited arch form expression effects.

### 2.6 Clinical bonding procedure

On clinical bonding, the patient's dentition is cleaned, pumiced, etched with phosphoric acid 37 %, and isolated. A primer is applied to the teeth and an orthodontic adhesive is applied to the bracket bases, then the indirect bonding trays are placed with firm pressure for a complete seat (Fig. 2d). The adhesive is light-cured for 40 seconds and the trays are carefully removed. When rigid trays are selected, carbide burs and silicone polishers (One Gloss, Shofu, Japan) are used to separate the occlusal and incisal parts, followed by the removal of the remaining parts surrounding the brackets.

After tray removal, intraoral scans may be taken to compare the actual bracket positions with the predetermined ones on setup models using 3D inspection software so that wrongly positioned brackets are detected.

## 3 Case 1 presentation

### 3.1 History

A 30-year-old Asian female patient presented with chief complaints of anterior crowding in the lower arch and mild protrusion of the upper incisors. Her medical and dental histories were non-contributory.

### 3.2 Assessment

**Fig. 4:**
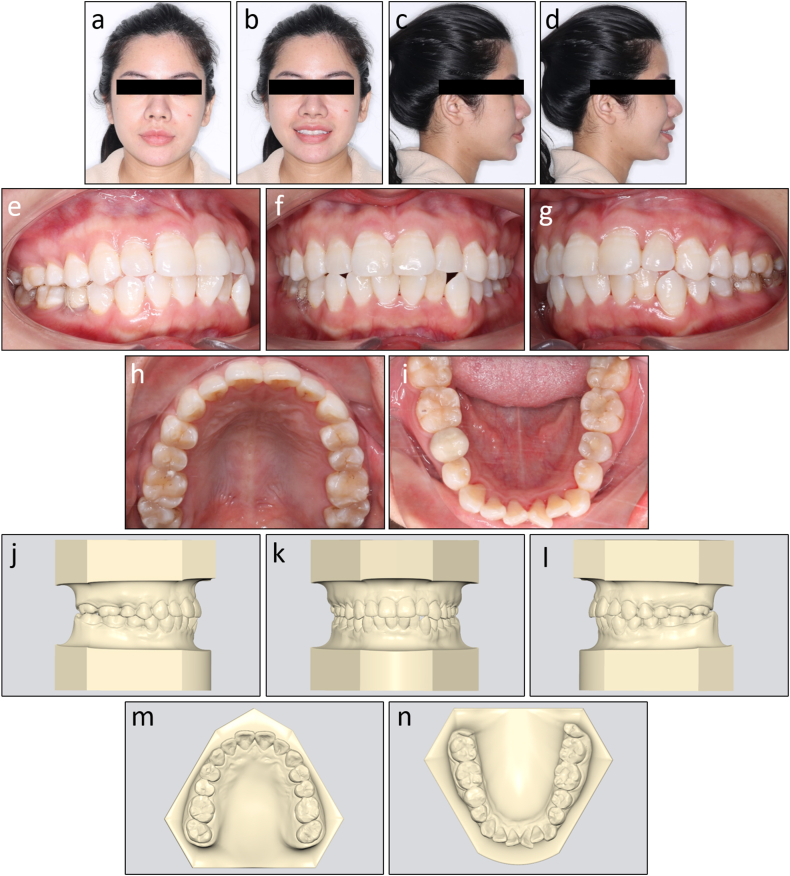
Pretreatment facial, intraoral photographs, and study models of Case 1. (a) Frontal. (b) Frontal smiling. (c) Lateral. (d) Lateral smiling. (e) Right occlusion. (f) Anterior occlusion. (g) Left occlusion. (h) Upper arch. (i) Lower arch. (j) Model right occlusion. (k) Model anterior occlusion. (l) Model left occlusion. (m) Model upper arch. (n) Model lower arch.

On extraoral examination, she had a slightly convex profile and her chin deviated to the left (Fig. 4a–d). No signs of temporomandibular joint disorders were detected.

On intraoral examination, the patient had mild Class III canine and molar relationships on the left side and Class I canine and molar relationships on the right side (Fig. 4e–n). The mandibular second premolars were endodontically treated and the right one was restored with a ceramic crown. Her mandibular dental midline deviated 2 mm to the left compared to the upper and facial midline.

There was moderate crowding with an arch length discrepancy of 5.7 mm in the lower arch. An anterior crossbite presented on the maxillary left lateral incisor and mandibular left canine.

### 3.3 Diagnostic tests

**Fig. 5:**
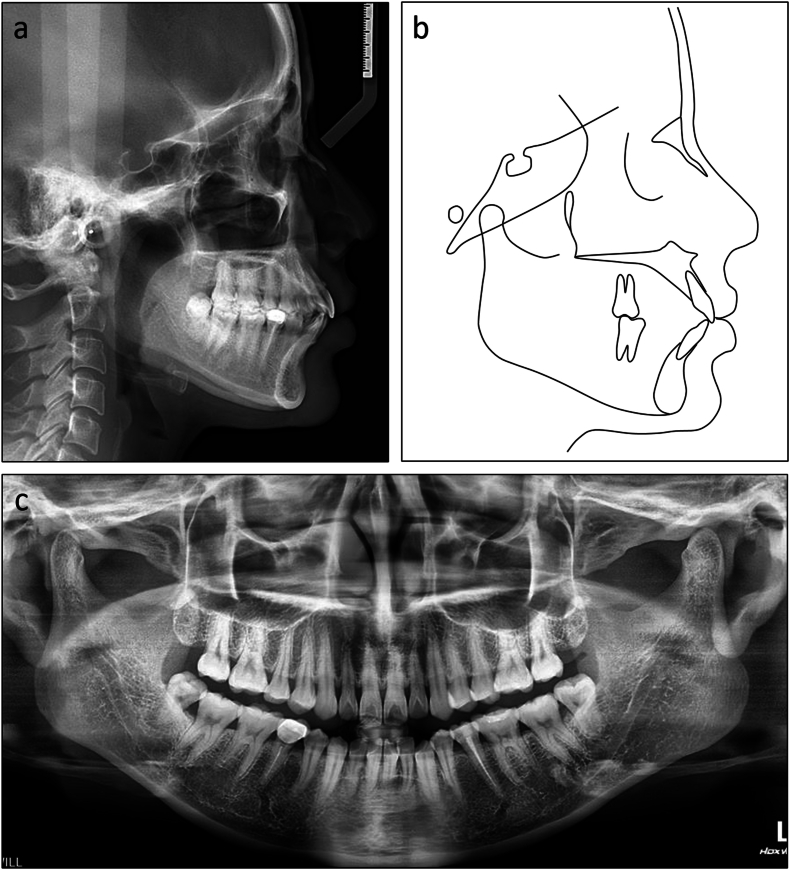
Pretreatment radiographs and cephalometric tracing of Case 1. (a) Cephalometric radiograph. (b) Cephalometric tracing. (c) Panoramic radiograph.

The lateral cephalometric evaluation revealed a Class I skeletal relationship and a normodivergent facial pattern with ANB 3.9°, Wits appraisal −0.5 mm, and maxillary mandibular angle 24.2° (Table 1). Both maxillary and mandibular incisors were proclined with U1 to maxillary plane 113.5°, L1 to mandibular plane 103.9°, and interincisal angle 118.4° (Fig. 5a and b). The panoramic radiograph confirmed the presence of all teeth except the maxillary third molars (Fig. 5c).

**Table 1:** Cephalometric variables of Case 1.

	Pretreatment	Posttreatment	Norms
SNA (°)	79.7	79.9	82 ± 3
SNB (°)	75.8	75.7	79 ± 3
ANB (°)	3.9	4.2	3 ± 1
SN to maxillary plane (°)	12.2	12.1	8 ± 3
Wits appraisal (mm)	−0.5	0.4	−0.3 ± 2.7
U1 to maxillary plane (°)	113.5	106.8	108 ± 5
L1 to mandibular plane (°)	103.9	92.7	92 ± 5
Interincisal angle (°)	118.4	135.0	133 ± 10
Maxillary mandibular plane (°)	24.2	25.5	27 ± 5
Upper anterior face height (mm)	51.1	50.8	54 ± 5
Lower anterior face height (mm)	62.3	62.7	65 ± 5
Lower anterior face height ratio (%)	54.9	55.3	55 ± 2
L1 to A-Pog (mm)	6.9	4.3	1 ± 1
Lower lip to E-plane (mm)	1.2	−1.3	−2 ± 1
Nasolabial angle (°)	96.6	101.4	95 ± 5

ANB, A point, nasion, B point; L1, lower central incisor; SN, sella nasion; SNA, sella nasion point A; SNB, sella nasion point B; U1, upper central incisor.

### 3.4 Treatment alternatives and plan

The main treatment objectives included relieving crowding in the lower arch without proclining the lower incisors, reducing the upper incisor proclination, correcting the Class III molar and canine relationships, and aligning the lower dental midline.

The first treatment option was to extract the mandibular third molars followed by distalizing the entire lower arch with skeletal anchorage and interproximal stripping. The second option was to extract the second premolars in both arches combined with Class III elastics. However, due to the patient's quite balanced profile and moderate crowding, the closure of remaining spaces after the alignment stage possibly leads to loss of lip support [13]. Considering these factors, the first option was chosen.

### 3.5 Treatment progress

First, the patient was referred to an oral surgeon for extraction of all third molars. The orthodontic treatment was started by bonding all teeth with 0.018 × 0.025 inch self-ligating lingual brackets (LinPass, Medico, Korea) except the mandibular left central incisor. Flexible 3D-printed indirect bonding trays were used. The alignment archwire sequence was 0.012-inch, 0.016-inch, and 0.016 × 0.022-inch nickel-titanium. After gaining adequate space with open coil springs, the mandibular left central incisor's lingual bracket was bonded with a 3D-printed individual transfer jig. The patient initially experienced mild tongue soreness and some pronunciation difficulties, but adaptation successfully took place within the first few weeks of treatment.

After 5 months of treatment, the archwires reached 0.016 × 0.022-inch stainless steel in both arches. The interproximal reduction was performed from the left to right first molars in both arches with a reduction amount of approximately 0.2 mm on each tooth side using a rotary diamond disc. The proximal surfaces were thoroughly polished and remineralized with topical fluoride after reduction.

**Fig. 6:**
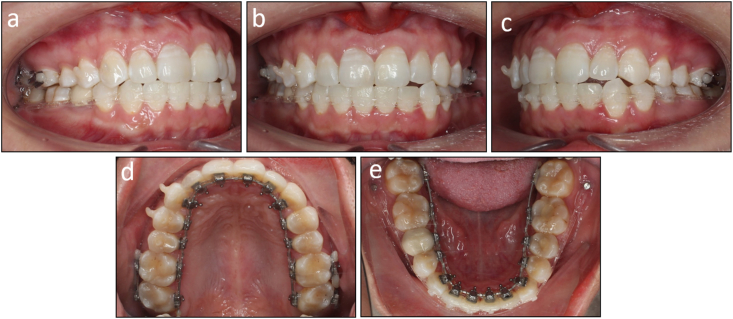
Distalization of the entire lower arch with mini-screws. (a) Right occlusion. (b) Anterior occlusion. (c) Left occlusion. (d) Upper arch. (e) Lower arch.

One month later, two mini-screws (diameter, 2.0 mm; length 12 mm; Medico, Korea) were inserted in the mandibular buccal shelf. Power chains were applied from the mini-screws to the lower anterior teeth with a force of 200 g to distalize the entire lower arch (Fig. 6a–e). After 3 months of distaliztion, the Class I canine and molar relationship was obtained. Some first-order bends were placed in the finishing stage. The total treatment time was 11 months. No bracket failure occurred throughout the treatment progress. Fixed retainers were bonded in both arches, along with clear retainers for nighttime wear.

### 3.6 Treatment results

**Fig. 7:**
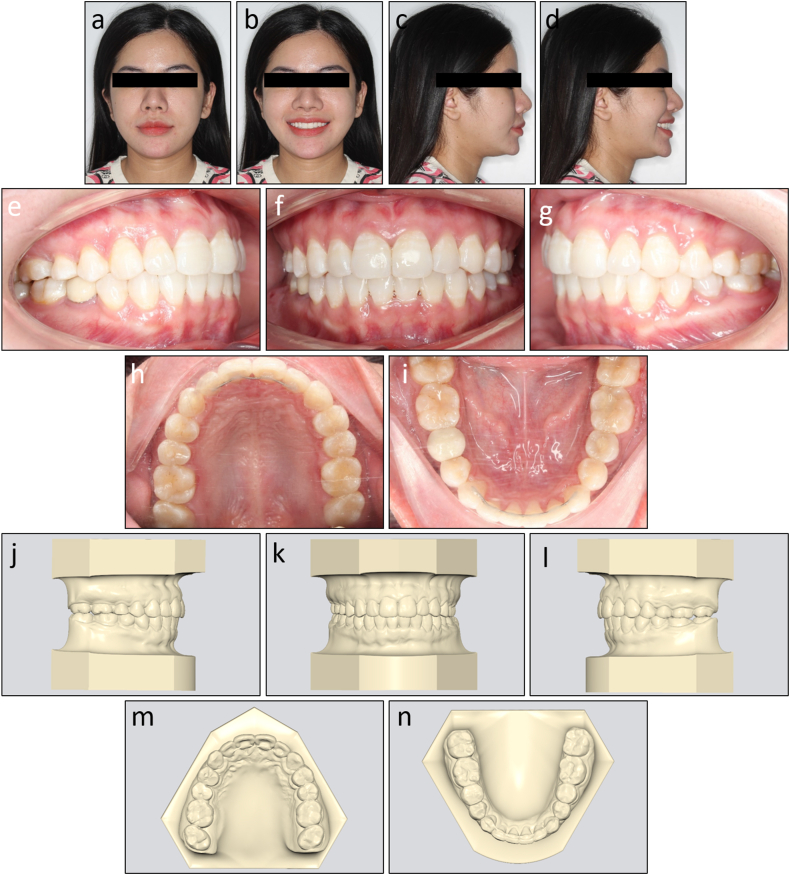
Post-treatment facial, intraoral photographs, and study models of Case 1. (a) Frontal. (b) Frontal smiling. (c) Lateral. (d) Lateral smiling. (e) Right occlusion. (f) Anterior occlusion. (g) Left occlusion. (h) Upper arch. (i) Lower arch. (j) Model right occlusion. (k) Model anterior occlusion. (l) Model left occlusion. (m) Model upper arch. (n) Model lower arch.

**Fig. 8:**
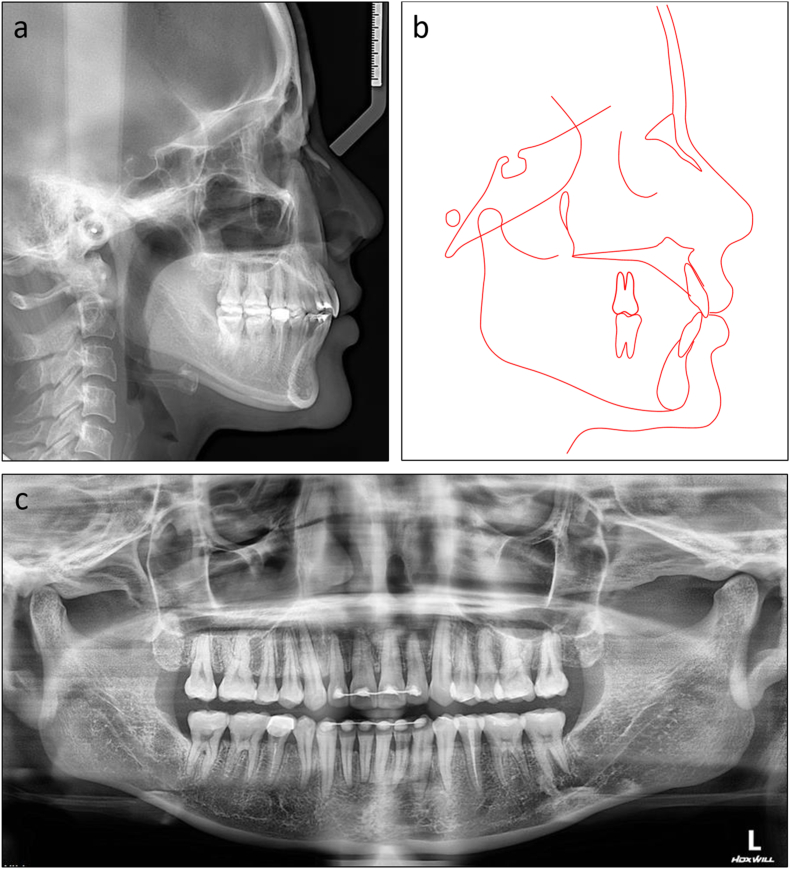
Post-treatment radiographs and cephalometric tracing of Case 1. (a) Cephalometric radiograph. (b) Cephalometric tracing. (c) Panoramic radiograph.

**Fig. 9:**
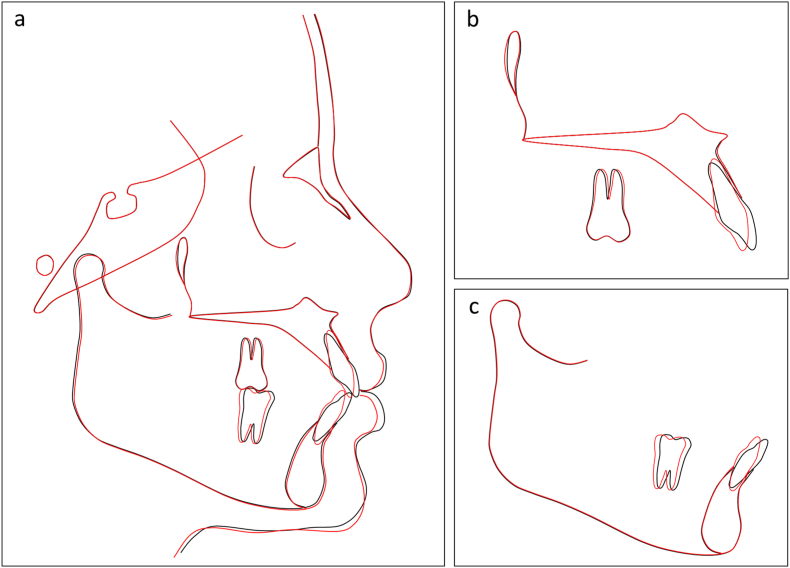
Overall and regional cephalometric superimpositions, before (black) and after treatment (red) of Case 1. (a) Overall superimposition. (b) Maxillary superimposition. (c) Mandibular superimposition.

**Fig. 10:**
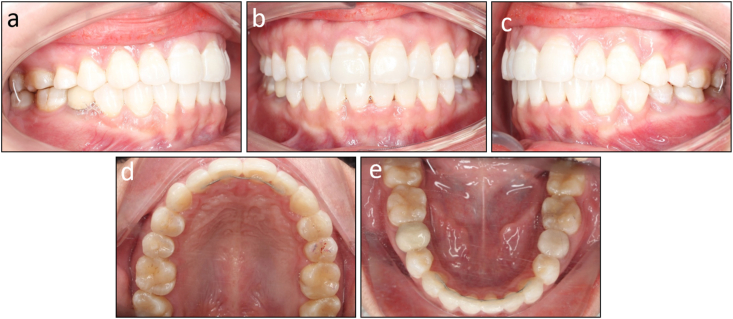
Post-retention intraoral photographs of Case 1. (a) Right occlusion. (b) Anterior occlusion. (c) Left occlusion. (d) Upper arch. (e) Lower arch.

The post-treatment records showed that treatment objectives were achieved with a well-aligned dentition, Class I canine and molar relationship on both sides, normal overjet and overbite, and coincided dental midlines (Fig. 7a–n). The lateral cephalometric evaluation showed that the inclination of the maxillary and mandibular incisors was reduced with U1 to maxillary plane 106.8°, L1 to mandibular plane 92.7°, and interincisal angle 135.0° (Fig. 8a and b). The panoramic radiograph showed good root parallelism without root resorption (Fig. 8c). The cephalometric superimpositions showed the retraction of the upper and lower incisors, the distalization of the mandibular molars, and the reduction of the lower lip prominence (Fig. 9a–c). The patient was reevaluated 12 months after treatment, and the results were found to be stable (Fig. 10a–e).

## 4 Case 2 presentation

### 4.1 History

A 31-year-old Asian male patient presented with a chief complaint of mouth protrusion. He was in overall good health and had no history of systemic diseases. His maxillary left central incisor had a history of dental trauma but no symptom was reported.

### 4.2 Assessment

**Fig. 11:**
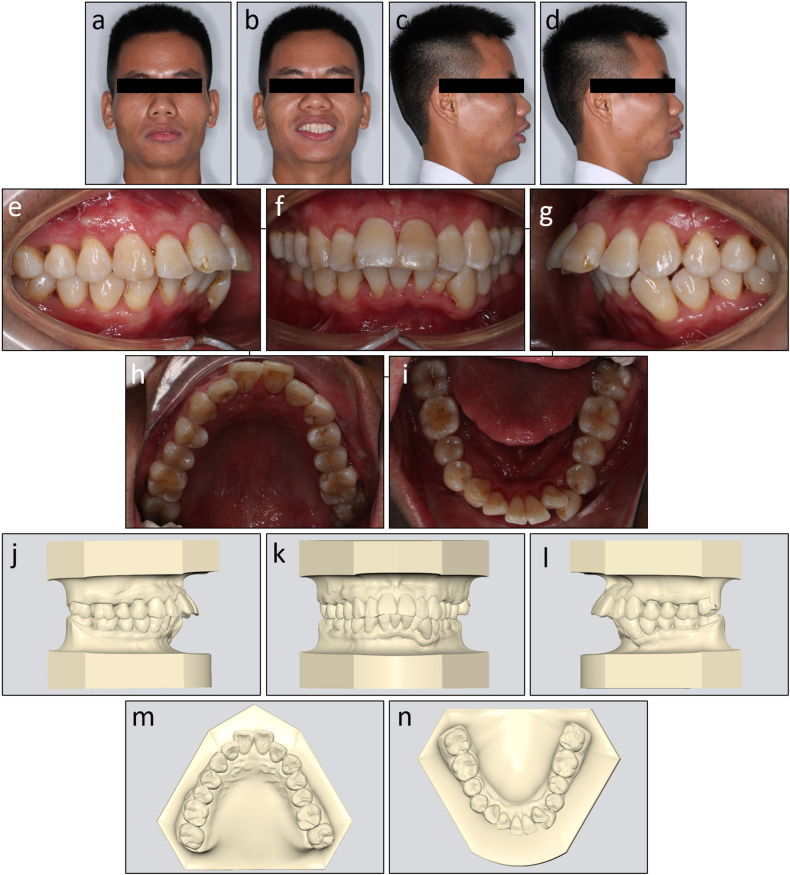
Pretreatment facial, intraoral photographs, and study models of Case 2. (a) Frontal. (b) Frontal smiling. (c) Lateral. (d) Lateral smiling. (e) Right occlusion. (f) Anterior occlusion. (g) Left occlusion. (h) Upper arch. (i) Lower arch. (j) Model right occlusion. (k) Model anterior occlusion. (l) Model left occlusion. (m) Model upper arch. (n) Model lower arch.

On extraoral examination, the patient had a convex profile, protrusive and incompetent lips, and a retruded chin (Fig. 11a–d). His mandible deviated to the left. No signs of temporomandibular joint disorders were observed.

On intraoral examination, the patient had Class I molar relationships and Class II end-on canine relationships on both sides (Fig. 11e–n). His lower dental midline deviated 4 mm to the left side. The arch length discrepancies were 3.4 mm and 7.1 mm in the upper and lower arch, respectively. He had an excessive overjet of 7.1 mm and a deep overbite of 4.3 mm. The maxillary left central incisor responded within a normal threshold with an electric pulp test.

### 4.3 Diagnostic tests

**Fig. 12:**
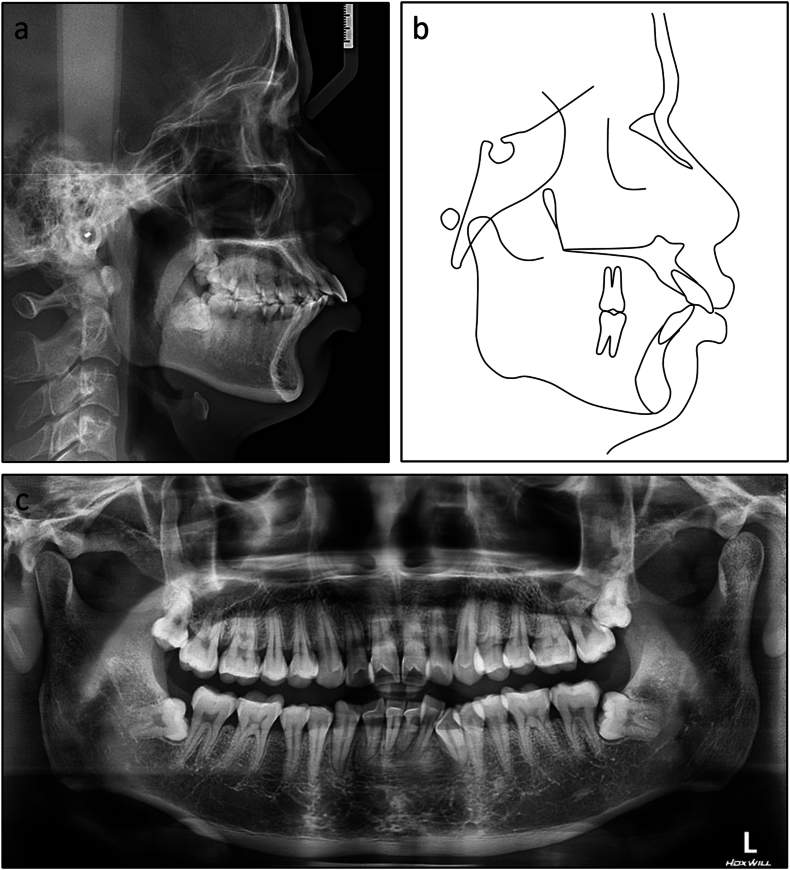
Pretreatment radiographs and cephalometric tracing of Case 2. (a) Cephalometric radiograph. (b) Cephalometric tracing. (c) Panoramic radiograph.

On lateral cephalometric assessment, the patient had a skeletal Class II relationship and a hypodivergent facial pattern with ANB 7.5°, Wits appraisal 7.1 mm, and maxillary mandibular angle 18.2° (Table 2). Both maxillary and mandibular incisors were severely proclined with U1 to maxillary plane 125.4°, L1 to mandibular plane 117.0°, and interincisal angle 99.4° (Fig. 12a and b). The panoramic radiograph showed the presence of all teeth with impacted mandibular third molars (Fig. 12c).

**Table 2:** Cephalometric variables of Case 2.

	Pretreatment	Posttreatment	Norms
SNA (°)	84.7	84.4	82 ± 3
SNB (°)	77.2	77.4	79 ± 3
ANB (°)	7.5	7.0	3 ± 1
SN to maxillary plane (°)	10.6	10.4	8 ± 3
Wits appraisal (mm)	7.1	2.7	−0.3 ± 2.7
U1 to maxillary plane (°)	125.4	99.4	108 ± 5
L1 to mandibular plane (°)	117.0	100.0	92 ± 5
Interincisal angle (°)	99.4	142.8	133 ± 10
Maxillary mandibular plane (°)	18.2	17.8	27 ± 5
Upper anterior face height (mm)	51.3	51.2	54 ± 5
Lower anterior face height (mm)	63.5	63.1	65 ± 5
Lower anterior face height ratio (%)	55.3	55.2	55 ± 2
L1 to A-Pog (mm)	6.5	4.3	1 ± 1
Lower lip to E-plane (mm)	7.9	0.8	−2 ± 1
Nasolabial angle (°)	91.9	99.4	95 ± 5

ANB, A point, nasion, B point; L1, lower central incisor; SN, sella nasion; SNA, sella nasion point A; SNB, sella nasion point B; U1, upper central incisor.

### 4.4 Treatment alternatives and plan

The main treatment objectives involved retracting the upper and lower incisors to relieve lip protrusion, aligning and leveling the dental arches, reducing the excessive overjet and deep overbite, and correcting the lower dental midline deviation.

Considering the skeletal Class II relationship, the first treatment option was a combined orthognathic-orthodontic plan in which the orthodontic decompensation would involve extracting the first premolars in both arches and space closure with maximum anchorage in the lower arch and moderate anchorage in the upper arch. The second treatment option was an orthodontic camouflage plan, also involving the extraction of the first premolars in both arches, but with space closure using maximum anchorage in the upper arch and moderate anchorage in the lower arch. Because the patient refused orthognathic surgery, the second treatment plan was chosen.

### 4.5 Treatment progress

The patient elected to have all third molars removed and was referred to an oral surgeon for the extractions. The orthodontic treatment was initiated by bonding all teeth with 0.018 × 0.025 inch double-slotted lingual brackets (ADB, Medico, Korea) except the mandibular left canine. Rigid 3D-printed indirect bonding trays were used. Two months later, the first premolars were extracted, and power chains were applied from the first molars to the canines to partially retract the canines, creating space for the incisor alignment. When space was adequately obtained, the mandibular left canine lingual bracket was bonded with a 3D-printed individual transfer jig.

**Fig. 13:**
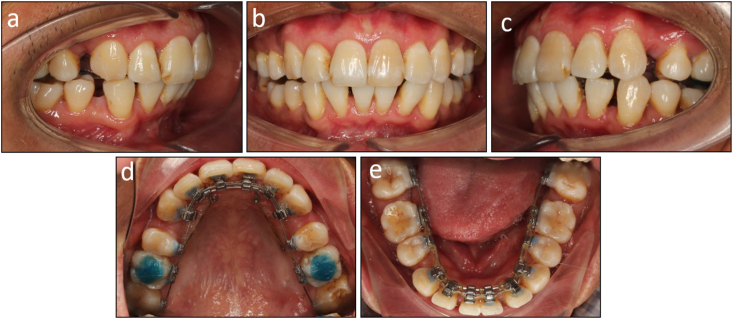
Space closure with sliding mechanics. (a) Right occlusion. (b) Anterior occlusion. (c) Left occlusion. (d) Upper arch. (e) Lower arch.

After six months of treatment, space closure was initiated on the upper arch with the main archwire of 0.016 × 0.022-inch stainless steel with 10° lingual root torque. Two miniscrews (diameter, 1.6 mm; length 10 mm; Medico, Korea) were inserted in the palatal alveolar bone between the maxillary second premolars and first molars for absolute anchorage. Power chains were applied from the mini-screws to crimpable hooks with a force of 150 g on each side to retract the upper anterior teeth (Fig. 13a–e). The space closure on the lower arch was started three months later with the main archwire of 0.016 × 0.022-inch stainless steel.

After 12 months of space closure, three-fourths of the first premolar extraction spaces were closed with torque loss in upper incisors. A 0.017 × 0.025 inch stainless steel archwire with 15° lingual root torque was inserted on the upper arch to correct the torque loss [14]. Vertical elastics were applied on both sides to settle the buccal occlusion, along with Class II elastics on the left to correct the left-sided Class II dental relationship and lower dental midline deviation. No wire bending was needed in the finishing stage. The total treatment time was 26 months. Only one bracket failure occurred on the left lateral incisor during the first month of treatment and was rebonded with a rigid individual transfer jig. After bracket removal, lingual fixed retainers were bonded in both arches, along with clear retainers for nighttime wear.

### 4.6 Treatment results

**Fig. 14:**
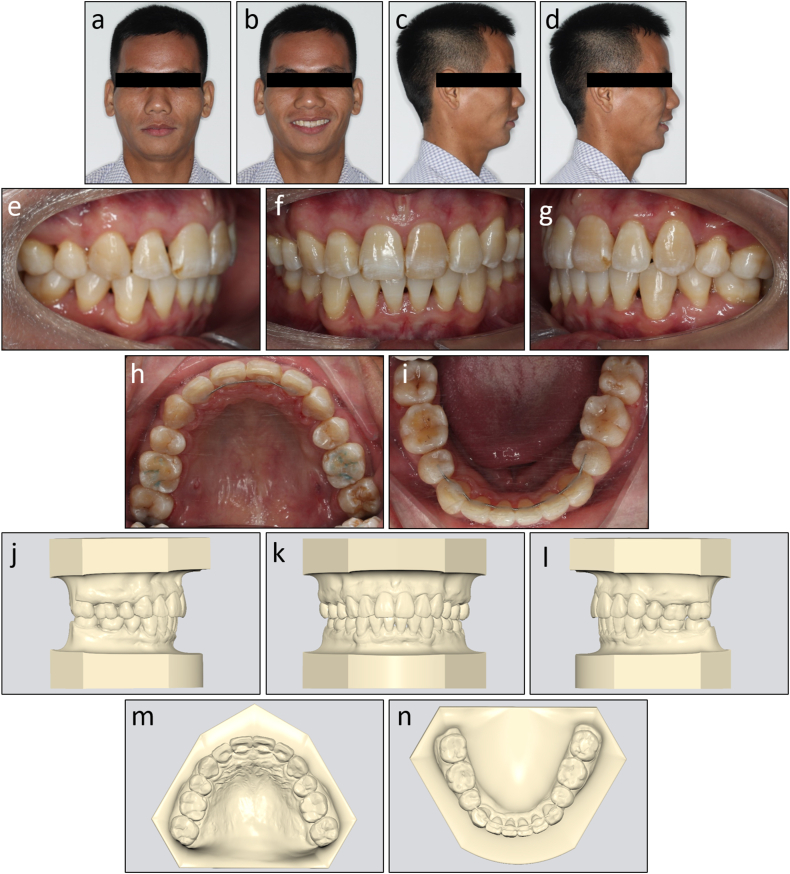
Post-treatment facial, intraoral photographs, and study models of Case 2. (a) Frontal. (b) Frontal smiling. (c) Lateral. (d) Lateral smiling. (e) Right occlusion. (f) Anterior occlusion. (g) Left occlusion. (h) Upper arch. (i) Lower arch. (j) Model right occlusion. (k) Model anterior occlusion. (l) Model left occlusion. (m) Model upper arch. (n) Model lower arch.

**Fig. 15:**
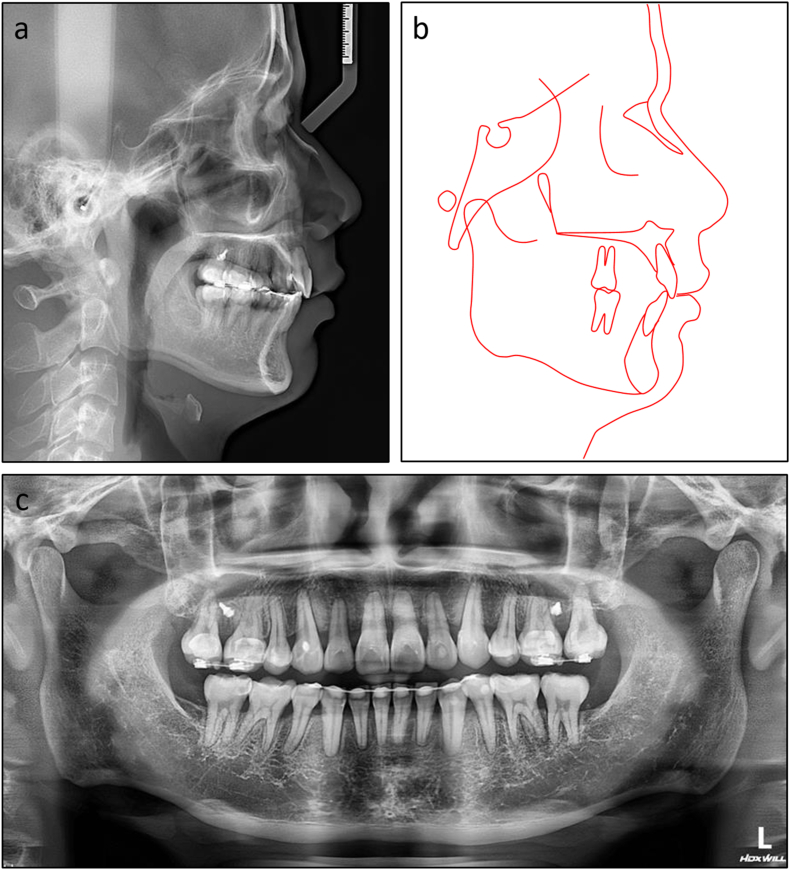
Post-treatment radiographs and cephalometric tracing of Case 2. (a) Cephalometric radiograph. (b) Cephalometric tracing. (c) Panoramic radiograph.

**Fig. 16:**
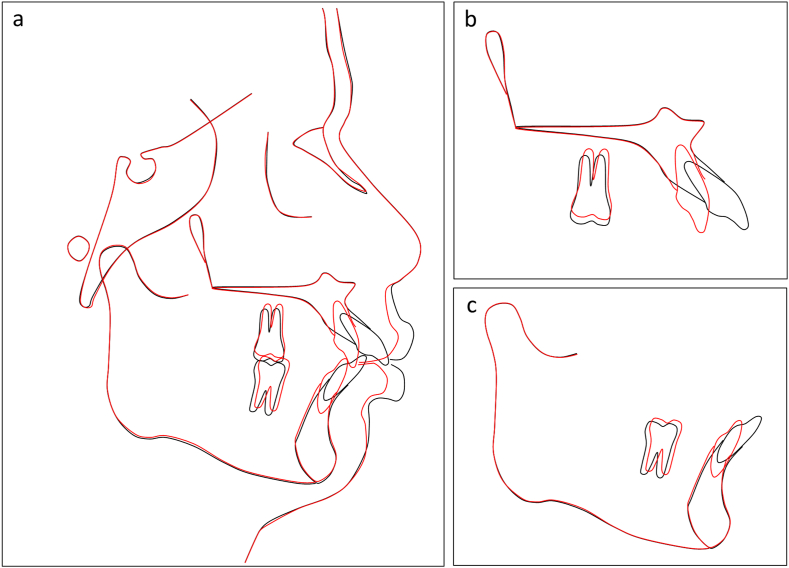
Overall and regional cephalometric superimpositions, before (black) and after treatment (red) of Case 2. (a) Overall superimposition. (b) Maxillary superimposition. (c) Mandibular superimposition.

**Fig. 17:**
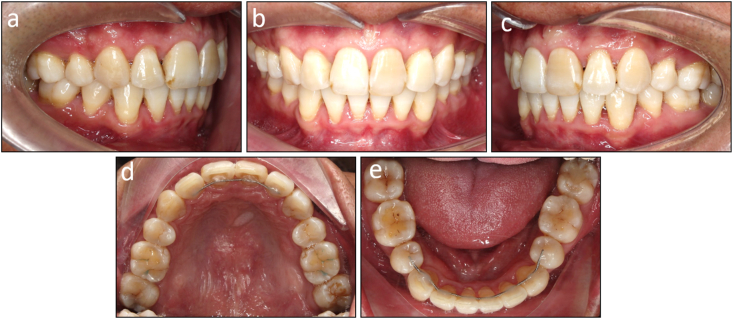
Post-retention intraoral photographs of Case 2. (a) Right occlusion. (b) Anterior occlusion. (c) Left occlusion. (d) Upper arch. (e) Lower arch.

The post-treatment photographs showed that the facial aesthetics and occlusion were significantly improved with a more balanced profile and well-aligned dentition (Fig. 14a–n). Class I canine and molar relationships were obtained bilaterally with normal overjet and overbite. The lower dental midline deviation was almost corrected. The lateral cephalometric evaluation confirmed the improvement of skeletal Class II relationship with ANB 7.0° and Wits appraisal 2.7 mm. Both upper and lower incisors were significantly retracted with U1 to maxillary plane 99.4°, L1 to mandibular plane 100.0°, and interincisal angle 142.8° (Fig. 15a and b). The panoramic radiograph showed that adequate root parallelism was obtained (Fig. 15c). However, mild root resorption was observed on the upper incisors, possibly due to the large movement amounts required for these teeth. Additionally, alveolar bone reduction was observed, requiring periodontal supervision after orthodontic treatment [15]. The cephalometric superimpositions confirmed the controlled tipping of the upper and lower incisors, the absolute anchorage of the upper molars, and the improvement of the lip projection (Fig. 16a–c). The 12-month post-retention photographs showed that the treatment results were stable (Fig. 17a–e). The patient was satisfied with the treatment results and the invisibility of the lingual appliances.

## 5 Discussion

At present, the procedures of fabricating lingual bracket indirect bonding trays and transfer jigs are usually performed in the laboratory with associated costs and delivery times [3,[16], [17], [18], [19]]. With the advancement of 3D-printing technology, orthodontists have the possibility to design and fabricate labial bracket indirect bonding trays independently from the laboratory [20,21]. However, the step-by-step techniques for in-office 3D-printed lingual bracket indirect bonding trays and transfer jigs are not described in the literature.

The traditional analog technique for fabricating lingual bracket transfer jigs includes silicone impressions, study model pouring, die cutting, orthodontic setup creation, lingual bracket bonding with a full-size archwire, and transfer jig fabrication. These steps are highly technique-sensitive and necessitate the involvement of experienced technicians. Additionally, it is challenging to accurately measure and adjust the tip and torque prescription values with the analog orthodontic setup.

In this analog technique, lingual brackets are usually transferred individually since they cannot be moved along with the teeth to the initial malocclusion state. When bonding individual jigs, there is less anatomy reference which may cause the jigs to displace mesially or distally leading to transfer inaccuracies [18,22]. Moreover, in this technique, the transfer jigs have to be reusable for rebonding failed brackets during treatment. Consequently, the retention between the lingual bracket and its lodgement is intentionally adjusted to be somewhat loose, necessitating the use of an elastic module to secure the bracket [16]. This loose retention may also affect the transfer accuracy.

An in-office technique for the fabrication of lingual bracket transfer jigs was described in the research of Anh et al. In this technique, ideal setup models with bracket guides are 3D printed, and individual bracket transfer jigs are created with flowable composites based on the Kommonbase method [23]. However, the bracket guides only facilitate the correct positioning of bracket bases. Transfer errors may occur when bracket bodies are incorrectly welded on bracket bases. Furthermore, the removal of bracket guides after adhesive light curing is time-consuming and uncomfortable for the patient.

Another in-office technique for the manufacture of vacuum-formed lingual bracket indirect bonding trays using 3D-printed models was also presented [7]. In this technique, an intermediate step of fabricating 3D-printed working models with resin brackets is necessary which may lead to errors. Additionally, the vacuum-formed trays may be deformed during removal from the 3D-printed models. This technique has been demonstrated to have high transfer accuracy in mesiodistal, buccolingual, occlusogingival directions, and rotation. However, tip and torque are transferred with less accuracy [22]. Furthermore, the rebonding of failed brackets is challenging as the vacuum-formed trays do not have a passive fit when only one tooth is involved.

The present digital workflows for designing and fabricating 3D-printed indirect bonding trays and transfer jigs for lingual brackets offer several advantages. Firstly, these digital workflows involve fewer steps, possibly resulting in a more time-efficient and less technique-sensitive process compared with the analog method. Secondly, the indirect bonding trays are directly printed, eliminating the need for immediate fabrication steps like bracket guides or vacuum-forming required in the previous digital methods. This can potentially reduce material consumption and improve bracket transfer accuracy. Furthermore, clinicians can achieve independence from laboratories thanks to the availability of free 3D design software [24]. However, it's crucial to maintain strict control over 3D printers, resins, and printing parameters to ensure high-accuracy printing for well-fitting transfer trays and accurate bracket positioning.

Indirect bonding trays of labial brackets are usually 3D-printed from flexible resin to facilitate tray removal after light curing [5,6]. However, individual transfer jigs for lingual brackets are generally made from rigid resin to achieve a more precise fit [16,18]. Currently, there is not enough clinical data to compare the accuracy of the two resin types. From the author's experience, rigid bonding trays are more passively seated and easier to print and clean. However, they require more chairside time for tray removal. In contrast, flexible bonding trays are more dependent on finger pressure [5]. In vivo studies should be conducted to compare the effectiveness and efficiency of these resin types. Furthermore, the comparison should be made between different tray designs such as fully versus partially enclosed lodgements and individual versus segmented tooth spans. The accuracy of 3D-printed trays should also be compared with vacuum-formed and silicone trays.

A possible limitation of this case series is its small sample size of only two patients, which may limit the generalizability of the results to the broader population. Additionally, the transfer accuracy of the bracket positions from the digital setup to the patients' dentition was not evaluated in this study. Future research with larger sample sizes and objective measurements of transfer accuracy is warranted.

## 6 Conclusion

With the advancement of 3D-printing technology, orthodontists can design and fabricate 3D-printed indirect bonding trays and transfer jigs for lingual brackets independently from the laboratory. The presented case reports showed that 3D-printed indirect bonding trays could transfer lingual brackets accurately and contribute to good treatment results. Additional studies with large sample sizes should be conducted to compare the effectiveness and efficiency of 3D-printed trays with other tray types.

## Ethics statement

Informed consents were acquired from the patients and the patients consented to the publishing of all images and clinical data included in the manuscript.

## Funding

This work received no external funding.

## Data availability

All data generated or analyzed during this study are included in this manuscript.

## CRediT authorship contribution statement

**Viet Anh Nguyen:** Writing – review & editing, Writing – original draft, Software, Methodology, Data curation, Conceptualization.

## Declaration of competing interest

The authors declare that they have no known competing financial interests or personal relationships that could have appeared to influence the work reported in this paper.
